# Ensembl Genomes 2013: scaling up access to genome-wide data

**DOI:** 10.1093/nar/gkt979

**Published:** 2013-10-25

**Authors:** Paul Julian Kersey, James E. Allen, Mikkel Christensen, Paul Davis, Lee J. Falin, Christoph Grabmueller, Daniel Seth Toney Hughes, Jay Humphrey, Arnaud Kerhornou, Julia Khobova, Nicholas Langridge, Mark D. McDowall, Uma Maheswari, Gareth Maslen, Michael Nuhn, Chuang Kee Ong, Michael Paulini, Helder Pedro, Iliana Toneva, Mary Ann Tuli, Brandon Walts, Gareth Williams, Derek Wilson, Ken Youens-Clark, Marcela K. Monaco, Joshua Stein, Xuehong Wei, Doreen Ware, Daniel M. Bolser, Kevin Lee Howe, Eugene Kulesha, Daniel Lawson, Daniel Michael Staines

**Affiliations:** ^1^The European Molecular Biology Laboratory, The European Bioinformatics Institute, The Wellcome Trust Genome Campus, Hinxton, Cambridgeshire, CB10 1SD, UK, ^2^Wellcome Trust Sanger Centre, The Wellcome Trust Genome Campus, Hinxton, Cambridgeshire, CB10 1SA, UK, ^3^Cold Spring Harbor Laboratory, 1 Bungtown Rd, Cold Spring Harbor, NY 11724, USA and ^4^USDA-ARS, Cornell University, Ithaca, NY, 14853, USA

## Abstract

Ensembl Genomes (http://www.ensemblgenomes.org) is an integrating resource for genome-scale data from non-vertebrate species. The project exploits and extends technologies for genome annotation, analysis and dissemination, developed in the context of the vertebrate-focused Ensembl project, and provides a complementary set of resources for non-vertebrate species through a consistent set of programmatic and interactive interfaces. These provide access to data including reference sequence, gene models, transcriptional data, polymorphisms and comparative analysis. This article provides an update to the previous publications about the resource, with a focus on recent developments. These include the addition of important new genomes (and related data sets) including crop plants, vectors of human disease and eukaryotic pathogens. In addition, the resource has scaled up its representation of bacterial genomes, and now includes the genomes of over 9000 bacteria. Specific extensions to the web and programmatic interfaces have been developed to support users in navigating these large data sets. Looking forward, analytic tools to allow targeted selection of data for visualization and download are likely to become increasingly important in future as the number of available genomes increases within all domains of life, and some of the challenges faced in representing bacterial data are likely to become commonplace for eukaryotes in future.

## OVERVIEW AND ACCESS

Ensembl Genomes (http://www.ensemblgenomes.org) is organized as five sites, each focused on one of the traditional kingdoms of life: bacteria (specific URL http://bacteria.ensembl.org), protists, fungi, plants and (invertebrate) metazoa. Vertebrate metazoa are the focus of the Ensembl project (http://www.ensembl.org) (1); Ensembl Genomes provides a complementary set of interfaces for non-vertebrate species. Core data available for all species include genome sequence and annotations of protein-coding and non-coding genes; additional data include transcriptional data, polymorphisms and comparative analysis. Interactive access is provided through a web interface providing genome browsing capabilities: users can scroll through a graphical representation of a DNA molecule at various levels of resolution, seeing the relative locations of features—including conceptual annotations [e.g. genes, single nucleotide polymorphism (SNP) loci], sequence patterns (e.g. repeats) and experimental data (e.g. sequences and external sequence features mapped onto the genome)—supporting the primary annotations. Functional information is provided through direct curation, import from the UniProt Knowledgebase (2) or imputation from protein sequence [using the classification tool InterProScan (3)]. Users can download much of the data available on each page in a variety of formats, and tools exist for upload of (various types of) user data, allowing users to see their own annotation in the context of the reference sequence. DNA- and protein-based sequence search are also available.

The data are stored in a set of MySQL databases using the same schemas as those in use for the Ensembl project. Direct access to these is provided through a public MySQL server (mysql.ebi.ac.uk:4157; user ‘anonymous’) and additionally through well-developed Application Programming Interfaces (APIs) that provide an object-oriented framework for working with the data. Database dumps and common data sets (e.g. DNA, RNA and protein sequence sets and sequence alignments) can be directly downloaded in bulk via file transfer protocol (ftp://ftp.ensemblgenomes.org).

Ensembl Genomes data are also made available through a series of data warehouses, optimized around common (gene and SNP-centric) queries, using the BioMart data warehousing system (4). The BioMart framework provides a series of interfaces, including web-based query building tools, for each of the Ensembl Genomes (eukaryotic) domains (e.g. at http://plants.ensembl.org/biomart/martview) and a variety of other interfaces for interactive and programmatic access. BioMarts are not currently available for Ensembl Bacteria.

Ensembl Genomes is released 4–5 times a year, in synchrony with releases of Ensembl, using the same software as the corresponding Ensembl release. The overall suite of Ensembl Genomes interfaces mirrors the interfaces provided for vertebrate genomes in Ensembl, and allows users access to genomic data from across the tree of life in a consistent manner.

## A COLLABORATIVE MODEL FOR GENOME-SCALE DATA

The Ensembl Genomes project is driven by a number of domain-specific collaborations, each with a scientific community with its own focus of interest. By working in partnership with us, communities can benefit from a robust infrastructure and the integration of their data within a comprehensive service. These collaborations take a number of forms. In some domains, we work with our partners to develop a community-centric service, aimed at each community’s specific needs, but also mirror key data within the central Ensembl Genomes portal. Examples of such collaborations include VectorBase (http://www.vectorbase.org) (5), a resource for the genomes of invertebrate pathogens of human diseases; WormBase (http://www.wormbase.org) (6), which maintains resources for nematode genomes, especially the model species *Caenorhabditis elegans*; PomBase (http://www.pombase.org) (7), the model organism database for the fission yeast *Schizosaccharomyces pombe*; and PhytoPath (http://www.phytopathdb.org), a resource for plant pathogens, with a focus on fungi and oomycetes. In other domains, we collaborate more broadly with other integrative centers, with a goal of developing high-quality networks of interlinked resources through the sharing of common reference data and standards for interoperability. In the context of Ensembl Plants, for example, we work closely with the Gramene database (http://www.gramene.org) (8) and a number of leading European plant genomics and informatics centers through the transPLANT project (http://www.transplantdb.eu). In addition, we contribute to many community-driven projects to sequence, assemble and annotate particular genomes, and make the resulting data available through the Ensembl Genomes site.

Ensembl Genomes prioritizes data for incorporation, according to scientific importance. The criteria for priority treatment are first, data relevant to our specific collaborations; second, data from other major experimental species; and third, data from other species that provide local or remote evolutionary context for the priority species, and which are used to strengthen the comparative analysis provided in the site. For the first category of genomes, we actively work with our collaborators to produce the primary community-recognized annotation. For the second category, we supplement the reference annotation (often maintained by model organism databases or other similar resources) with additional high-value data sets. For several species in these two categories, we have constructed variation databases, which store genotypes, loci and phenotypes from large-scale genome-wide array-based and resequencing studies, and have made the data available through specialized graphical views and an SNP-centric BioMart. Variation data are sourced from dbSNP (9) or Database of Genomic Variants archive (10) where available, or otherwise directly from the data producers. For the third category of genomes, annotation is generally incorporated from the original submitters with only limited enhancement (for example, the annotation of non-coding genes, if absent in the original submission).

At the time of writing, there have been 10 releases of Ensembl Genomes since the previous report was published in this journal (11). The current release is release 20, made public in September 2013. In this time, there has been a significant increase in the content of all five Ensembl Genomes sites.

### Metazoa

Nineteen new genomes have been added, including the sponge *Amphimedon queenslandica*, the south and central American malarial mosquito *Anopheles darlingi*, the leaf-cutter ant *Atta cepahlotes*, the silkworm *Bombyx mori*, the water flea *Daphnia pulex*, the pacific oyster *Crassostrea gigas*, the owl limpet *Lottia gigantea*, the scuttle fly *Megaselia scalaris*, the centipede *Strigamia maratima*, the kissing bug *Rhodnius prolixus,* the red flour beetle *Tribolium casteneum*, the two-spotted spider mite *Tetranychus uriticae*, two annelid worms, two butterflies and three nematodes. Additional variation data (12,13) have been introduced for *Anopheles gambiae*, and new DNA-based comparative analysis has been added for nematodes.

### Plants

Twenty-two new genomes have been added, including Chinese cabbage (*Brassica rapa*); soy bean (*Glycine max*), barley (*Hordeum vulgare*), banana (*Musa acuminata*), barrel clover (*Medicago trunculata*), the club moss *Selagninella moellenorfii*, foxtail millet (*Setaria ilatica*), tomato (*Solanum lycopersicum*), potato (*Solanum tuberosum*), two species of rice, two diploid ancestors of hexaploid bread wheat and, as taxonomic outliers, two algal species. In addition, the preliminary genome assemblies, homeologous SNP calls and expressed sequence transcript (EST) sequences available for bread wheat have been aligned to the genomes of barley and *Brachypodium distachyon*, and a sequence search has been implemented against the EST sequences that visualized the results in the context of their alignments to these references. Variation databases have been provided for barley (14), maize (15), rice (16,17) and sorghum (18). The variation data set for *Arabidopsis thaliana* has been expanded to include additional data from the 1001 Genomes Project (19) and other work, including phenotypic data (20). Additional comparative alignments have been produced for cereal genomes.

### Fungi

Twenty-four new genomes have been added, including 20 plant pathogens (*Blumeria graminis, Botrytis cornerea*, *Fusarium oxysporum*, *Gaeumannomyces graminis*, *Glomerella gramincola*, *Leptosphaeria maculans*, *Melampsora larcini-populina*, *Microbotryum violaceum, Nectria haematococca*, *Puccinia triticina*, *Sclerotina sclerotiorum*, *Sporisorium reilanium*, *Trichoderma reesei*, *Ustilago maydis*, two species of *Gibberella*, two species of *Magnaporthe* and two species of *Pyrenophora*). Other species added include the human pathogen *Cryptococcus neoformans*, the truffle *Tuber melanosporum* and two additional yeast species. RNA-seq alignments (to the genome) have been added for *P. triticina*; EST alignments have been added for *Phaeosphaeria nodorum*, *S. pombe*, *T. melanosporum* and *Zymoseptoria tritici*; and new comparative genomic alignments have been added for certain *Pyrenophora* and yeast species. For phytopathogenic fungal (and protist) species, information about genes impacting on pathogenesis has been imported from the PHI-base database (21), and mutant and overexpression phenotypes are now represented in a color-coded form in the genome browser.

### Protists

Eleven new genomes, including those of several important plant and human pathogens, have been added: *Albugo laibachii*, *Entamoeba histolytica*, *Giardia lamblia*, *Guillardia theta*, *Hyaloperonspora arabidopsis*, *Leishmania major*, *Paramecium tetraurelia*, *Pythium ultimum*, *Tetrahymena thermophila*, *Toxoplasma gondii* and *Trypanosma brucei*. New DNA alignments have been provided for the ciliates, the Peronsoporales and the Trypanosomatidae. A variation database has been added for *Phytophthora infestans.*

### Bacteria

**Figure 1.**
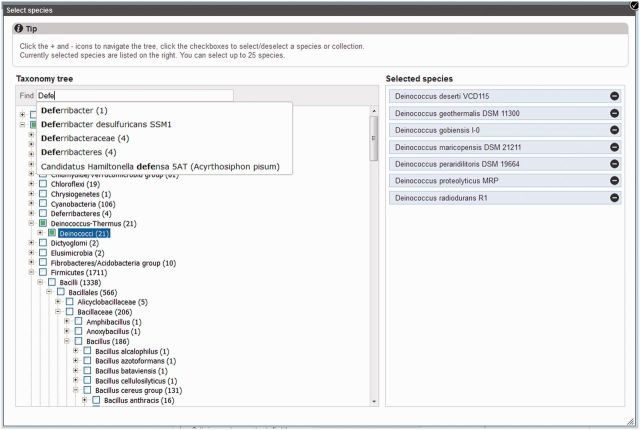
Species selection in Ensembl Bacteria. The figure shows the selection of a basket of genomes for use in a BLAST search. A tree-based navigation system allows the selection of defined portions of the taxonomy for use as library sequences. An autocomplete feature assists the location of particular genomes within the tree.

Ensembl Bacteria has been comprehensively expanded since release 17. Although previously the bacterial division of Ensembl had focused on a small number of selected clades, the division now contains all bacterial genomes that have been completely sequenced, annotated and submitted to the International Nucleotide Sequence databases (European Nucleotide Archive, GenBank and the DNA Database of Japan) (22), a total of 9089 genomes in the latest release. Additional information is incorporated from the UniProtKB, InterPro, information about operons from RegulonDB (23) and about reaction catalysts from Microme (http://www.micromedb.eu). To ensure that data within this expanded set remain discoverable, two new species selection mechanisms have been introduced into the portal, one using autocomplete and the other providing a taxonomically structured interface (illustrated in Figure 1). The latter also enables the restriction of (sequence and text) search to user-defined taxonomic segments. Additionally, the Ensembl Perl API has been extended with a new lookup module, allowing users to discover genomes matching their specifications (e.g. full or partial name-match, taxonomic identifier, nucleotide sequence accession) programmatically. Within the browser, an improved representation of transcripts and translations, capable of providing a correct representation of bacterial features (i.e. polycistronic transcripts and alternative translational initiation) has been introduced.

## IMPROVED TOOLS FOR DATA ACCESS

A number of improvements to the Ensembl infrastructure have been made during the past year, including the introduction of a scrollable browser and a new RESTful API (a language-agnostic supplement to the existing Perl API), whereas the range of data formats provided (for appropriate data types) via file transfer protocol has been expanded to include Genome File Format and Variant Call Format. A new fast sequence search, based on a back-end provided by the European Nucleotide Archive, has been introduced for all species alongside a Basic Local Alignment Search Tool (BLAST) server. A feature allowing portions of gene trees to be highlighted based on the existence of common annotation has been introduced. Support has been introduced for annotations comprising structured assemblies of ontology terms (e.g. for complex phenotypic description), and a new browser has been implemented for ontological terms, which depicts the ancestry of annotated terms and provides links through to BioMart to allow users to retrieve gene sets annotated with any term in the display. Finally, automatic display of remote files is now supported for any data file using any known synonym to identify the reference sequence on which the data is to be visualized.

## COMPARATIVE ANALYSIS

Extensive comparative analyses are performed between the sequences in Ensembl Genomes. Analyses include pairwise alignments between DNA sequences, using the tools LASTZ (24) and (for more diverged genomes) translated BLAT (25) combined with the use of the chain/net algorithm of Kent et. al (26). The number of these comparisons has increased and we now have 118 pairwise alignments. In Ensembl Plants, pairwise alignments are provided for rice against every other genome, *A. thaliana* against every other genome (except barley) and 14 other pairwise comparisons. In Ensembl Metazoa, comparisons are provided from *Drosophila melanogaster* to 11 other drosophilid species and 4 mosquitoes, for all pairwise combinations of *A. gambiae*, *A. darlingi*, *Aedes aegytptii* and *Culex quinquefasciatus*, from *C. elegans* to 8 other nematodes and from *Brugia malayi* to *Loa loa*. In Ensembl Fungi, all-against-all alignments are available in the *Aspergillus*, *Hypocreales*, *Pucciniales*, *Pyrenophora* clades and for *Saccharomyces cerevisiae* against *Ashyba gossypii*. In Ensembl Protists, DNA alignments are provided for each of three *Phytophthora species* against each other*.* No DNA-based comparisons are currently provided for bacterial species.

**Figure 2.**
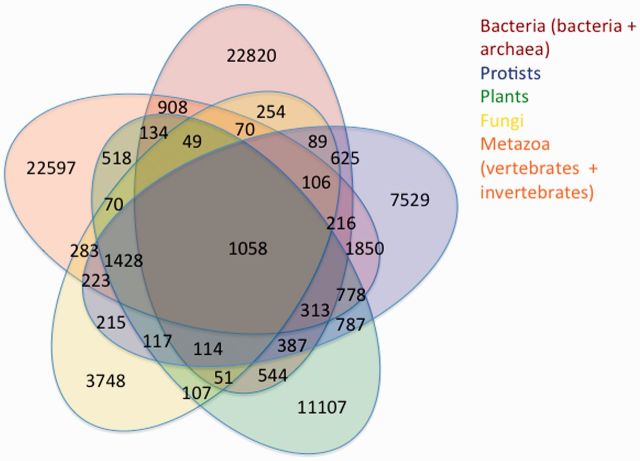
Taxonomic distribution of gene families in the pan-taxonomic comparative analysis in release 19 of Ensembl Genomes. Large numbers of families [defined by clustering according to the Ensembl Gene Trees algorithm (27)] are found only in one domain of life. However, families can be found spanning all combination of domains. The most overrepresented spans (compared with expectations based on the same proportion of families being covering each domain, but assuming the co-coverage of two domains is random) are (i) all five domains and (ii) all four non-bacterial domains; the most underrepresented spans are (i) bacteria and metazoa and (ii) bacteria, metazoa and fungi. For each family of related proteins, a gene tree is constructed and made available for visualization and download, estimating the evolutionary history of that family.

Protein alignments are used to reconstruct evolutionary trees for related genes using the Ensembl Compara Gene Trees pipeline (27). These are run for each eukaryotic domain and additionally for a representative selection of species from across the taxonomic space to identify widely conserved families and deep homologies between different evolutionary branches. In the current release, the pan-taxonomic database was constructed from the genomes of 12 chordates (11 vertebrates, plus *Ciona intestinalis*), 15 non-chordate metazoans, 7 plants, 7 fungi, 8 protists, 98 bacteria and 25 archaea. Genomes are chosen for inclusion according to a variety of criteria, including mutual taxonomic distance, number of recorded publications, prior inclusion in previous editions of the pan-taxonomic Compara and overlap with the reference proteome sets defined by the UniProt KB. In total, 79 005 gene trees have been constructed for a total of 1 070 325 proteins. Their distribution among the different taxonomic domains is shown in Figure 2. Bacterial proteins (from all included genomes) have additionally been grouped into families using the HAMAP (28) and Panther (29) resources.

## CEREALS: SERIOUSLY BIG GENOMES

**Figure 3.**
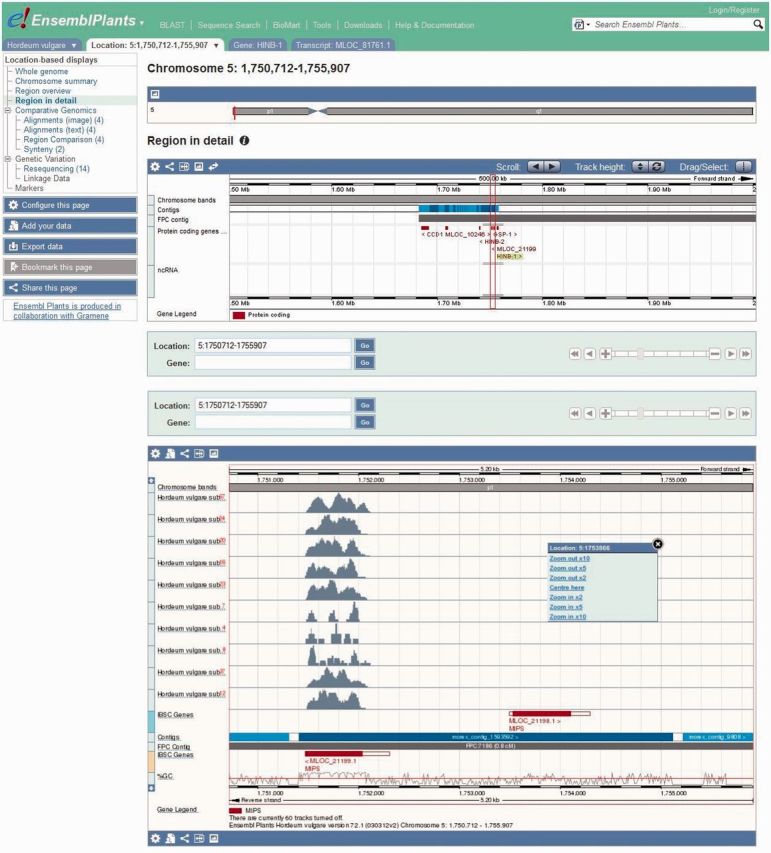
The barley genome represented in Ensembl Plants. The figure shows resequencing alignments from a number of cultivars against the reference cultivar Morex genome assembly and annotation for a sequenced contig given approximate chromosomal location through integration with the genetic map.

The genomes of several economically important crop species have not yet been completely sequenced owing to their large size and highly repetitive DNA. However, during the last year, early versions of the diploid barley genome (5 Gb) and the hexaploid bread wheat genome (16 Gb) have become available. Neither genome is yet available in a completely assembled form. The current barley genome assembly (14) consists of around 1.9 Gb of DNA in 612 267 contigs of over 200 bp, of which ∼ 400 Mb of which have been located on chromosome level using markers from extant physical and genetic maps. A total of 24 211 high-confidence protein-coding genes have been called, of which 64% are in anchored locations. The N50 is only 1405 bp, but the N50 of gene-containing scaffolds is much higher (8.4 Kb). Despite the fragmented nature of the genome, barley is represented conventionally in Ensembl Plants, with data shown at all levels from the karyotype through to comparative analysis and variation. In the absence of high-level scaffolding, approximate colocation of contigs to marker sequences located on the physical map is used to provide an approximation of the order and orientation of contigs at each chromosomal locus. Additionally, unanchored contigs have been grouped together in a synthetic ‘chromosome’ (consisting of the actual contigs with arbitrary gaps between them) to better fit the data model (and critically, to improve analysis times), and contigs of <200 nucleotides have been excluded from the database. In all other respects, the genome can be accessed in the same way as any of the better-assembled genomes in the resource. A typical view of the barley genome in Ensembl Plants is depicted in Figure 3.

The wheat genome assemblies published in late 2012 (30) are even more fragmentary with many contigs, even those in genic regions, less extensive than the genes themselves; thus, creation of accurate complete gene models is difficult. Therefore, in Ensembl Plants, we have presented the data in the form of alignments of the genomic contigs onto two better-assembled reference genomes, Brachypodium and barley. This enables the wheat sequences, and the homeologous variants (between the three wheat genomes) that have been identified, to be located in the context of full-length gene models predicted in these closely related species. Additionally, a set of 1.3 million wheat ESTs has been mapped onto the Brachypodium and barley genomes; and a sequence search facility provided against the wheat EST set that returns an alignment of the query sequence against the top-matching ESTs and additionally shows where those ESTs align to the genomes of these two related species.

## PERSPECTIVE AND PRIORITIES

Over the next 2 years, we anticipate that increasingly complete versions of the hexaploid bread wheat genome will emerge from the efforts of the International Wheat Genome Sequencing Consortium (http://www.wheatgenome.org/), allowing for a transition from the current limited representation of the genome to a more complete representation. As such, wheat will be the first polyploid genome to be fully represented in the Ensembl system. Although the size and repetitive nature of the genome is a challenge in terms of assembly and annotation, the Ensembl database schema and interface are flexible and should accommodate data from polyploid species with only minor modification. Each of the three genomes will be separately analyzed in the Ensembl comparative analysis pipelines, placing each genome separately within each gene tree and identifying homeologous regions of DNA sequence that will be displayed in an integrated stacked visualization. The existing representation of homeologous variants will be extended to show their functional consequence on gene models.

A more significant challenge lies in data discovery, as the number of available genomes and data sets continues to rise: how can users discover whether information that might be of interest to them exists in the system? We anticipate an increased need for data analysis tools not just as an end in themselves but also as a route of data access. Users will not necessarily start their analyses knowing which genomes or genes they wish to work on; instead, they might wish to ask questions of the complete data set to identify genomes that differ in terms of their gene content or genes whose presence/absence/copy number difference differentiates two genomes (for example, to determine the difference between pathogenic and non-pathogenic strains of related organisms or between competent and non-competent vectors). Likewise, variant features will also be studied in the context of their presence or absence in certain individuals/populations. Supporting these use cases will require rapid gene classification (including common occurrences of novel gene families identified in multiple species) and a high-performance data warehouse to support analysis of the data and help users identify the features of interest within the total data set. Another increasingly important use case is likely to be for the dynamic display of data on demand from archived analyses (e.g. sequence alignments, variant calls) selected on the basis of associated experimental metadata. Developing such tools will be a priority for Ensembl Genomes over the next years.

## FUNDING

UK Biosciences and Biotechnology Research Council [BB/I00I0077/1, BB/H531519/1, BB/F19793/1, BB/J017299/1, BB/J00328X/1, BB/I008071/1 to P.K.]; Wellcome Trust [090548/B/09/Z to P.K.]; Bill and Melinda Gates Foundation [OPPGD1491 to P.K.]; U.S. National Science Foundation [41686 IPGA Gramene to D.W.]; 7th Framework Programme of the European Union [contract numbers 228421, INFRAVEC; 222886-2, Microme; and 284496, transPLANT to P.K.]. Funding for open access charge: The European Molecular Biology Laboratory.

*Conflict of interest statement*. None declared.

## References

[1] Flicek P, Ahmed I, Amode MR, Barrell D, Beal K, Brent S, Carvalho-Silva D, Clapham P, Coates G, Fairley S (2013). Ensembl 2013. Nucleic Acids Res..

[2] The UniProt Consortium (2012). Update on activities at the Universal Protein Resource (UniProt) in 2013. Nucleic Acids Res..

[3] Hunter S, Jones P, Mitchell A, Apweiler R, Attwood TK, Bateman A, Bernard T, Binns D, Bork P, Burge S (2011). InterPro in 2011: new developments in the family and domain prediction database. Nucleic Acids Res..

[4] Kasprzyk A (2011). BioMart: driving a paradigm change in biological data management. Database.

[5] Megy K, Emrich SJ, Lawson D, Dialynas E, Hughes DS, Koscielny G, Louis C, Maccallum RM, Redmond SN VectorBase: improvements to a bioinformatics resource for invertebrate vector genomics. Nucleic Acids Res..

[6] Yook K, Harris TW, Bieri T, Cabunoc A, Chan J, Chen WJ, Davis P, de la Cruz N, Duong A, Fang R (2012). WormBase 2012: more genomes, more data, new website. Nucleic Acids Res..

[7] Wood V, Harris MA, McDowall MD, Rutherford K, Vaughan BW, Staines DM, Aslett M, Lock A, Bähler J, Kersey PJ (2011). PomBase: a comprehensive online resource for fission yeast. Nucleic Acids Res..

[8] Youens-Clark K, Buckler E, Casstevens T, Chen C, DeClerck G, Derwent P, Dharmawardhana P, Jaiswal P, Kersey P, Karthikeyan AS (2011). Gramene database in 2010: updates and extensions. Nucleic Acids Res..

[9] Sherry ST, Ward MH, Kholodov M, Baker J, Phan L, Smigielski EM, Sirotkin K (2001). dbSNP: the NCBI database of genetic variation. Nucleic Acids Res..

[10] Lappalainen I, Lopez J, Skipper L, Hefferon T, Spalding JD, Garner J, Chen C, Maguire M, Corbett M, Zhou G (2013). DbVar and DGVa: public archives for genomic structural variation. Nucleic Acids Res..

[11] Kersey PJ, Staines DM, Lawson D, Kulesha E, Derwent P, Humphrey JC, Hughes DS, Keenan S, Kerhornou A, Koscielny G (2012). Ensembl Genomes: an integrative resource for genome-scale data from non-vertebrate species. Nucleic Acids Res..

[12] Weetman D, Wilding CS, Steen K, Morgan JC, Simard F, Donnelly MJ (2010). Association mapping of insecticide resistance in wild anopheles gambiae populations: major variants identified in a low-linkage disequilbrium genome. PLoS One.

[13] Weetman D, Wilding CS, Steen K, Pinto J, Donnelly MJ (2011). Gene flow–dependent genomic divergence between anopheles gambiae M and S forms. Mol. Biol. Evol..

[15] Chia J-M, Song C, Bradbury PJ, Costich D, de Leon N, Doebley J, Elshire RJ, Gaut B, Geller L, Glaubitz JC (2012). Maize HapMap2 identifies extant variation from a genome in flux. Nat. Genet..

[16] McNally KL, Childs KL, Bohnert R, Davidson RM, Zhao K, Ulat VJ, Zeller G, Clark RM, Hoen DR, Bureau TE (2009). Genomewide SNP variation reveals relationships among landraces and modern varieties of rice. Proc. Natl Acad. Sci. USA.

[17] Zhao K, Wright M, Kimball J, Eizenga G, McClung A, Kovach M, Tyagi W, Ali ML, Tung C-W, Reynolds A (2010). Genomic diversity and introgression in O. sativa reveal the impact of domestication and breeding on the rice genome. PLoS One.

[18] Zheng L-Y, Guo X-S, He B, Sun L-J, Peng Y, Dong S-S, Liu T-F, Jiang S, Ramachandran S, Liu C-M (2011). Genome-wide patterns of genetic variation in sweet and grain sorghum (Sorghum bicolor). Genome Biol..

[19] Weigel D, Mott R (2009). The 1001 genomes project for *Arabidopsis thaliana*. Genome Biol..

[20] Atwell S, Huang YS, Vilhjálmsson BJ, Willems G, Horton M, Li Y, Meng D, Platt A, Tarone AM, Hu TT (2010). Genome-wide association study of 107 phenotypes in *Arabidopsis thaliana* inbred lines. Nature.

[21] Winnenburg R, Urban M, Beacham A, Baldwin TK, Holland S, Lindeberg M, Hansen H, Rawlings C, Hammond-Kosack KE, Köhler J (2008). PHI-base update: additions to the pathogen host interaction database. Nucleic Acids Res..

[23] Salgado H, Peralta-Gil M, Gama-Castro S, Santos-Zavaleta A, Muñiz-Rascado L, García-Sotelo JS, Weiss V, Solano-Lira H, Martínez-Flores I, Medina-Rivera A (2013). RegulonDB v8.0: omics data sets, evolutionary conservation, regulatory phrases, cross-validated gold standards and more. Nucleic Acids Res..

[24] Harris R (2007). Improved Pairwise Alignment of Genomic DNA.

[25] Kent WJ (2002). BLAT—the BLAST-like alignment tool. Genome Res..

[26] Kent WJ, Baertsch R, Hinrichs A, Miller W, Haussler D (2003). Evolution’s cauldron: Duplication, deletion, and rearrangement in the mouse and human genomes. Proc. Natl Acad. Sci. USA.

[27] Vilella AJ, Severin J, Ureta-Vidal A, Heng L, Durbin R, Birney E (2009). EnsemblCompara GeneTrees: complete, duplication-aware phylogenetic trees in vertebrates. Genome Res..

[28] Pedruzzi I, Rivoire C, Auchincloss AH, Coudert E, Keller G, de Castro E, Baratin D, Cuche BA, Bougueleret L, Poux S (2012). HAMAP in 2013, new developments in the protein family classification and annotation system. Nucleic Acids Res..

[29] Mi H, Muruganujan A, Thomas PD (2013). PANTHER in 2013: modeling the evolution of gene function, and other gene attributes, in the context of phylogenetic trees. Nucleic Acids Res..

[30] Brenchley R, Spannagl M, Pfeifer M, Barker GLA, D’Amore R, Allen AM, McKenzie N, Kramer M, Kerhornou A, Bolser D (2012). Analysis of the bread wheat genome using whole-genome shotgun sequencing. Nature.

